# Approaching Training-Practice Gaps After the Transition: A Practice Proposal for Supervision After Training

**DOI:** 10.3389/fmed.2022.881274

**Published:** 2022-05-06

**Authors:** Olle ten Cate, Robert P. Favier

**Affiliations:** ^1^Utrecht University Medical Center, Utrecht University, Utrecht, Netherlands; ^2^IVC Evidensia, Utrecht, Netherlands

**Keywords:** supervision, transitions, entrustable professional activities, entrustment decisions, self-assessment

## Abstract

Transitions within medical, veterinarian, and other health professional training, from classroom to workplace, between undergraduate, postgraduate, fellowship phases, and to unsupervised clinical practice, are often stressful. Endeavors to alleviate inadequate connections between phases have typically focused on preparation of learners for a next phase. Yet, while some of these efforts show results, they cannot obliviate transitional gaps. If reformulated as ‘not completely ready to assume the expected responsibilities in the next phase’, transitions may reflect intrinsic problems in a training trajectory. Indeed, the nature of classroom teaching and even skills training for example, will never fully reflect the true context of clinical training. In various stages of clinical training, the supervision provided to trainees, particularly medical residents, has increased over the past decades. This addresses calls for enhanced patient safety, but may inadequately prepare trainees for unsupervised practice. Transitions often evolve around the question how much support or supervision incoming trainees or junior professionals require. We propose to consider receiving incoming trainees and new employees in clinical workplaces with a conversation about required supervision for discrete tasks, or entrustable professional activities (EPAs). EPAs lend themselves for the question: “At what level of supervision will you be able to carry out this task?”. This question can be answered by both the trainee or junior employee and the supervisor or employer and can lead to agreement about specified supervision for a defined period of time. We expect that this “supported autonomy tool” could alleviate stress and enhance continued development after transitions.

## Introduction

Transitions in the health professions education (medical, veterinarian, and other health professions) have been a frequent topic of debate and research (1–6). These transitions are moments in which individuals experience discontinuity in their professional life space, forcing to respond by developing new behaviors or changing a life space in order to cope with the new situation (7). Significant transitions in the continuum of medical education include the step from classroom education to clinical training, from undergraduate to postgraduate training and from postgraduate training to unsupervised practice (8). Sometimes this implies a change in legal status. A license to practice after medical, veterinary, dental, nursing, or other health professions training comes along with more formal responsibilities and expectations, and so does postgraduate certification.

Over history, medical students and residents have often reported transitions from pre-clinical to clinical education and from training to unsupervised practice to be stressful (9–11). Even the transitions caused by short rotations within postgraduate medical training have been reported to be stressful, potentially harming personal wellbeing as well as patient safety (12). Similarly, in other health professions transitions have been reported to be stressful (13–16). The causes of stress regard not only physiological and practical needs that must be addressed, but also new health care practice, community, role, responsibilities and level of autonomy, all recently summarized in a model named “Bates' hierarchy of contextual competence” (13). New-to-setting nurses can experience a tremendous amount of stress, feeling frustrated, lost, or angry (14). Veterinary graduates, licensed for independent practice, do not always feel ready for practice, which causes stress (15), and program directors in surgery have been reported to observe gaps in trainee preparedness for residency or for fellowship (16).

While accurate historical comparisons are hard, there are some indications that postgraduate trainees have become less prepared for practice across the past decades. This may be a side effect of efforts to increase patient safety in teaching hospitals. Both supervision and number of trainees have increased, while duty hours and trainees' autonomy have decreased (17). This has, no doubt, increased patient safety in teaching hospitals. However, in the US and likely in other countries, many tasks and activities in previous decades done by residents are now routinely done by attendings, leaving residents with less experience and creating bigger transitional gaps when unsupervised practices commences (17–19). Transitions to postgraduate medical training or practice can lead to stress, burnout, switches or termination of education, and leaving the profession (20–22).

It can be argued that transitions have both an affective, emotional component and a cognitive and psychomotor component.

## Transitions and the Affective, Emotional Component: The Impostor Phenomenon

Emotions resulting from transitions can be seen as an impostor phenomenon (IP). The word imposter is defined as “one who assumes a false character, or passes himself off as some one other than he really is” (23). It explains the impostor phenomenon: “the persistent inability to believe that one's success is deserved or has been legitimately achieved as a result of one's own efforts or skills” (23). A substantial percentage of medical students and residents seem to experience IP (24, 25) unrelated to their actual competence (26). While most IP studies focus on associations with gender, self-esteem, comorbidities and treatment, some studies show a decrease with age (24). A transition to a new context, with unknown colleagues and unfamiliar routines, particularly after completion of training, or with a new license or certification, imposes expectations that may feel uncomfortable, and particularly if that context includes more experienced colleagues (27). It is unsurprising that new team members experience at least a temporary imposter phenomenon, and fear the moment that they can no longer mask their “incompetence”, an emotion that may fade over time. Emotional support directly after transitions may be warranted. The phenomenon becomes a syndrome if it persists unduly for a long time.

## Transitions and the Cognitive and Psychomotor Component: Transfer of Learning and the Zone of Proximal Development

Two theory-based perspectives may shed light on the cognitive and psychomotor component of transitioning to a new context. One stems from the concept of “transfer”, and one is the concept of “zone of proximal development”. Transfer relates to the ability to apply what was learned in one context (classroom, or a specific working context) in a different context. Transfer is a core purpose of education, but the extent to which transfer happens, can be enhanced, or can be predicted has been a topic of debate (28). Transitions in health professions education usually imply transfer of what was learned when a change of context occurs. Recently, fuelled by COVID-19, a call for a redefinition of medical competence voiced the proposal to include 'capability' (or adaptive expertise; the ability to adapt to new contexts and challenges), rather than just fixed competencies (29, 30). This requires transfer. Salomon and colleagues introduced the distinction between near and far transfer (between very similar or dissimilar contexts), and low road and high road transfer (requiring little or large mental effort and mindful abstraction of principles that can be applied in a different context than learned). Far, high road transfer is not impossible, but cannot always be assumed to happen, and transfer depends on the 'distance' or similarity between contexts (31). To prepare learners for transfer, practicing in various contexts, avoiding rote learning and stimulating understanding, deliberate practice and mindful abstraction of concepts has been recommended, (32) but if new contexts or tasks differ substantially from the context of learning or tasks learned, transfer can simply not be assumed to happen. While adequate unsupervised performance in a similar but new context by qualified individuals may be expected to some extent, but there is a limitation to distance between familiar and unfamiliar contexts in which this will be successful. Vygotsky defined the Zone of Proximal Development for learners as “the distance between one's actual developmental level as determined by independent problem solving and the level of potential development as determined through problem solving under adult guidance or in collaboration with more capable peers (p86)” (33). In other words, transitions that imply such transfer, even within that space, require some external support or supervision.

## The Role of Entrustable Professional Activities

While entrustable professional activities (EPAs) were proposed to define the activities postgraduate trainees should be able to be entrusted with before completion of training, and thus marking the transition to unsupervised practice (34), they have also been conceived to ease other transitions, such as from undergraduate to postgraduate medical education (35, 36) which seems effective (37). EPAs, as units of professional practice (tasks) that may be entrusted to a learner to execute unsupervised, once they have demonstrated the required competence (38), can facilitate transitions in three ways. One way is the provision of clarity of expectations of tasks that learners will face after a training period. The second facilitating factor is that qualifications for the unsupervised practice of various relevant EPAs do not come all at once at the same time. EPAs can stimulate a gradual increase of clinical responsibilities and autonomy, one by one, as soon as a learner is ready to bear the responsibility for those respective EPAs; each on their own time. Finally, with EPAs the transfer of responsibility is not just a matter of all or none; responsibility is translated in decreasing levels of supervision (direct, indirect, distant or no supervision).

## Easing Transitions Before and After the Fact

Much of the efforts of decreasing transitional problems for students has been put at the front-end of the transition (39). The preparation of students for anticipated transitions has dealt with focused clinical skills training, capstone courses (40) and curricular integrations, in particular vertical integration (41). Vertical integration prioritizes terminal objectives of a program in early curricular phases, such as teaching basic sciences from the perspective of patient care, but also building a gradual increase of clinical responsibilities through legitimate peripheral participation of juniors in a community of practice (42, 43). However, even with maximum preparation, transitions will always confront learners with a new context and the need to adapt.

After transitions educators usually have no influence on mitigating transitional difficulties. In some cases, employers, with no educational mission, must deal with deficiencies that become visible after the transition (15). Orientation or induction periods for new employees can help, but the educational commitment is limited. Educational handovers (providing information about a learner's past performance for a next context) has been suggested to improve transitions, but is a sensitive topic of debate (44). While qualified professionals are assumed to be ready for the tasks they have been trained in, the need for supervision after transitions is increasingly acknowledged. As education should include continuous professional development (45, 46), it is a sensible idea to look at what receiving phases can do to alleviate problems of transitions. Ilgen et al. have stressed the need for supervision in an early autonomy phase when unsupervised practice comes along with uncertainty and called this “supported independence” (47). Transitions often evolve around the question how much support or supervision incoming trainees or junior professionals require (15, 48, 49).

## Supervision for EPAs After Formal Qualification

Becoming qualified and permitted to perform EPAs with indirect or no supervision requires a decision that, until recently, was made implicitly and formalized with the completion of training or the awarding of a license to practice. With the advent of EPAs and individualized training programs, programs and their clinical competency committees make formal decisions, or statements of awarded responsibility (STARs) about EPAs, granting more autonomy to trainees, before the end of their training (50, 51). The most salient decision is to qualify a trainee ready for unsupervised practice. The practice of granting this qualification has fueled questions of generality. If, for example, a gynecology program, during one rotation in one hospital, would provide this STAR-testimony for the EPA “uncomplicated child delivery”, should other hospitals accept this STAR or not? If a community hospital has carefully made such a summative entrustment decision, can or should a tertiary university hospital in a next rotation count on that qualification and withhold supervision? We believe that a check of applicability of this qualification in every new context is warranted, whether within the training period or in a new employment environment. That check may be short, but can also lead a decision to provide a period of supervision. While all recommendations to define an EPA stress that it is important to provide full clarity of what the activity entails (with specifications and limitations) (52), context-dependent disparities may remain (13). In addition, the recommended full description of EPAs includes an expiration date (52). Critical EPAs that have not been practiced for years, may require renewed supervision, for example when a senior clinician is asked to run a night shift to replace a senior resident and is expected to perform tasks that were once familiar but not anymore.

## A Practical Proposal to Monitor Supervision Requirements Upon Transitions

A new trainee or new employee, embarking on a clinical rotation, a residency or a new job in a less familiar context may use the framework of EPAs to discuss with their new supervisor or employer what will be reasonable expectations for the initial phase. We envision EPAs as a mechanism to make the expectations of trainee and supervisor or employer within a new context explicit. These expectations may be framed as desired (or required) levels of supervision. The dialogue in the first day after the transition can focus on a list of EPAs, with the question, for each, how much supervision the new trainee or employee desires or expects. An agreement may arise after negotiation. Such dialogue is likely to alleviate uncomfortable feelings and stress, caused by a lack of clarity of expectations. It can also stimulate a learning curve of the trainee, inspired by a restricted time of supervision for EPAs that should have actually been mastered before.

**Figure 1:**
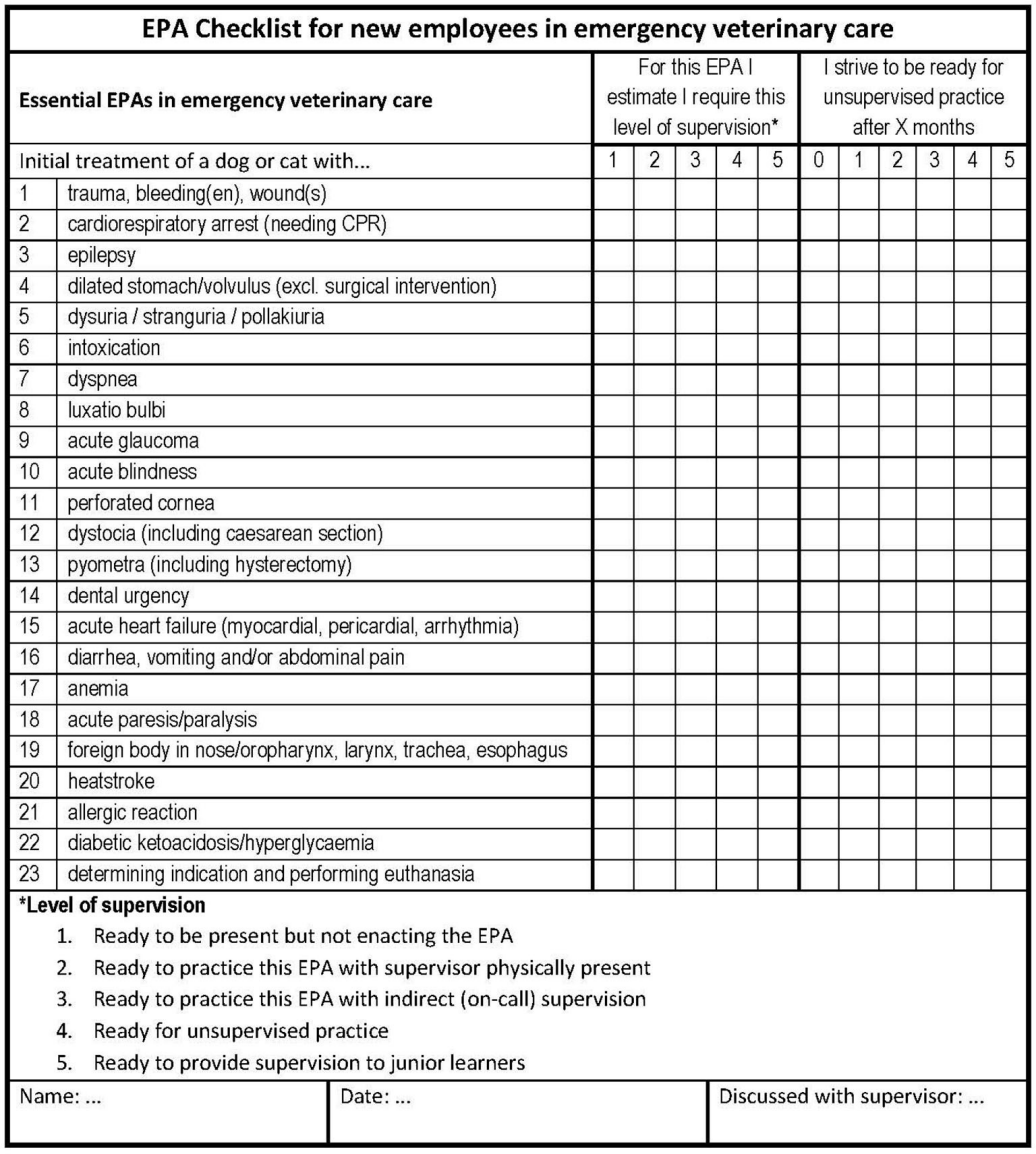
Proposed tool to manage supervision after transition (example from veterinary medicine).

Figure 1 shows an example of this approach, inspired by a current study among veterinarians after graduation, who start with an appointment at an emergency veterinary clinic. The study currently investigates the effects of job clarification with EPAs on job satisfaction, stress, mental wellbeing, and attrition among new employees in such clinics in the Netherlands.^1^

This approach aligns well with the recommendations to “encourage progressive independence by offering a sliding scale of decreasing supervision alongside demonstrating increasing trust (both globally and for specific tasks)” and to “make postgraduate trainees aware of the psychological impact of actual responsibility (including the process of their own identity formation) once they move up a level of training or into consultancy” (39).

## Discussion

Our proposal requires application, monitoring, and validation in practice, with adequate research designs. Several questions may be addressed in such studies. One question, for instance, is how well new trainees or employees are able to evaluate their own competence. Self-assessment is difficult (53). The so-called Dunning-Kruger effect suggests that high performers generally under-estimate themselves, while low performers over-estimate themselves (54). Recently, critics have pointed at a misinterpreted statistical regression that would explain Dunning-Kruger as an artifact (55), but even if so, self-assessment remains difficult without reliable feedback. The approach we propose, however, could correct wrong self-images in practice, and help to recalibrate self-reported competence and improve self-assessment.

Another construct to measure is self-efficacy. Self-efficacy for professional tasks (EPAs) is known to correlate with job satisfaction (56) and is fed, according to Bandura's theory, by the experience of successes, adequate verbal feedback, and vicarious experience (57). The mere presence of someone you can turn to for confirmation, questions and feedback, a supervisor, in an early phase in a new work environment can be critical to managing stressful situations.

Our expectation is that a focus on expected supervision for well described tasks after transition will help to alleviate the uncomfortable feelings and stress, caused by working in a less familiar environment, because it provides clarity and grip on this environment by making expectations explicit. If requests for supervision were foreseen in a signed document, that provision is likely to lower the threshold of asking for supervision.

As educational programs in the health professions are increasingly organized by entrustable professional activities, these units of professional practice lend themselves for the question after graduation: “At what level of supervision will you be able to carry out this task?”. This question can be answered by both trainee or employee (depending on the next career stage) and supervisor or employer and can even lead to an agreement about supervision for a defined period of time. We expect this approach to alleviate stress and enhance continued development after transitions. In addition, a feedback-loop mechanism may be created to inform a prior educational program about the general preparedness of learners in new contexts. This will require a reporting mechanism, probably aggregated across multiple individuals and with confidentiality conditions, but it would potentially be a great educational quality improvement method.

Following Ilgen et al. (46) and Sawatsky et al. (58), the document could be called a “supported autotomy tool”, as an instrument of self-determination, that could be added to a professional's personal dynamic portfolio across a life time (45). While in early phases educational handovers are a responsibility of teachers and the institution, this tool is owned by the individual to remind to reflect on one's own developing competence and occasional need for support in new contexts.

## Data Availability Statement

The original contributions presented in the study are included in the article/supplementary material, further inquiries can be directed to the corresponding author/s.

## Author Contributions

The conceptual idea for te approach described in this paper was from RF. The first draft of the manuscript was written by OtC. Several iterations of the manuscript were reviewed, commented and redacted by both authors. Both authors contributed to the article and approved the submitted version.

## Funding

Utrecht University Open Access Fund has covered the author processing costs.

## Conflict of Interest

RF was employed by IVC Evidensia. The remaining author declares that the research was conducted in the absence of any commercial or financial relationships that could be construed as a potential conflict of interest.

## Publisher's Note

All claims expressed in this article are solely those of the authors and do not necessarily represent those of their affiliated organizations, or those of the publisher, the editors and the reviewers. Any product that may be evaluated in this article, or claim that may be made by its manufacturer, is not guaranteed or endorsed by the publisher.

## Footnotes

^1^Favier RP, Bok HGJ, Ramaekers SPJ, ten Cate O. Task clarification and wellbeing in veterinary emergency care: a survey study of the effects of reframing the work as EPAs with desired supervision levels. Report in preparation.

## References

[1] Franzen D Kost A Knight C. Mind the gap: the bumpy transition from medical school to residency. J Grad Med Educ. (2015) 7:678–80. 10.4300/JGME-D-15-00413.1 26692989 PMC4675432

[2] Teunissen PW Westerman M. Junior doctors caught in the clash: the transition from learning to working explored. Med Educ. (2011) 45:968–70. 10.1111/j.1365-2923.2011.04052.x 21916936

[3] Westerman M Teunissen PW Fokkema JPI Van Der Vleuten CPM Scherpbier AJJA Siegert CEH. The transition to hospital consultant and the influence of preparedness, social support, and perception: A structural equation modelling approach. Med Teach. (2013) 35:320–7. 10.3109/0142159X.2012.735381 23527864

[4] Favier RP ten Cate O Duijn C Bok HGJ. Bridging the gap between undergraduate veterinary training and veterinary practice with entrustable professional activities. J Vet Med Educ. (2021) 48:136–8. 10.3138/jvme.2019-0051 32149590

[5] Teunissen PW Westerman M. Opportunity or threat: The ambiguity of the consequences of transitions in medical education. Med Educ. (2011) 45:51–9. 10.1111/j.1365-2923.2010.03755.x 21155868

[6] O'Brien BC. What to Do about the Transition to Residency? Exploring problems and solutions from three perspectives. Acad Med. (2018) 93:681–4. 10.1097/ACM.0000000000002150 29419551

[7] Westerman M. Mind The Gap. The Transition to Hospital Consultant. Doctoral Dissertation. Amsterdam: Free University (2012). p. 1–155.

[8] Wijnen-Meijer M Burdick W Alofs L Burgers C Ten Cate O. Stages and transitions in medical education around the world: Clarifying structures and terminology. Med Teach. (2013) 35. 10.3109/0142159X.2012.746449 23360484

[9] Prince KJAH Van de Wiel MWJ Scherpbier AJJA Van der Vleuten CPM Boshuizen HPA. A qualitative analysis of the transition from theory to practice in undergraduate training in a PBL-medical school. Adv Heal Sci Educ. (2000) 5:105–16. 10.1023/A:1009873003677 12386467

[10] Peters S Clarebout G van Nuland M Aertgeerts B Roex A. A qualitative exploration of multiple perspectives on transfer of learning between classroom and clinical workplace. Teach Learn Med. (2018) 30:22–32. 10.1080/10401334.2017.1339605 28753068

[11] Walker LG Haldane JD Alexander DA. A medical curriculum: evaluation by final-year students. Med Educ. (1981) 15:377–82. 10.1111/j.1365-2923.1981.tb02418.x 7329363

[12] Bernabeo EC Holtman MC Ginsburg S Rosenbaum JR Holmboe ES. Lost in transition: the experience and impact of frequent changes in the inpatient learning environment. Acad Med. (2011) 86:591–8. 10.1097/ACM.0b013e318212c2c9 21436668

[13] Teunissen PW Watling C Schrewe B Asgarova S Ellaway R Myers K. Contextual Competence: how residents develop competent performance in new settings. Med Educ. (2021) 55:1100–9. 10.1111/medu.14517 33630305 PMC8451833

[14] Chicca J Bindon S. New-to-setting nurse transitions: a concept analysis. J Nurses Prof Dev. (2019) 35:66–75. 10.1097/NND.0000000000000530 30829909

[15] Duijn C Bok H Ten Cate O Kremer W. Qualified but not yet fully competent: perceptions of recent veterinary graduates on their day-one skills. Vet Rec. (2020) 186. 10.1136/vr.105329 31767696

[16] Mattar SG Alseidi AA Jones DB Jeyarajah DR Swanstrom LL Aye RW. General surgery residency inadequately prepares trainees for fellowship: Results of a survey of fellowship program directors. Ann Surg. (2013) 258:440–7. 10.1097/SLA.0b013e3182a191ca 24022436

[17] Halpern SD Detsky AS. Graded autonomy in medical education–managing things that go bump in the night. N Engl J Med. (2014) 370:1086–9. 10.1056/NEJMp1315408 24645941

[18] Dacey RG Nasca TJ. Seniorization of tasks in the academic medical center: a worrisome trend. J Am Coll Surg. (2019) 228:299–302. 10.1016/j.jamcollsurg.2018.11.009 30481550

[19] George BC Dunnington GL DaRosa DA. Trainee autonomy and patient safety. Ann Surg. (2018) 267:820–2. 10.1097/SLA.0000000000002599 29166357

[20] Bustraan J Dijkhuizen K Velthuis S Van Der Post R Driessen E Van Lith JMM. Why do trainees leave hospital-based specialty training? A nationwide survey study investigating factors involved in attrition and subsequent career choices in the Netherlands. BMJ Open. (2019) 9:1–8. 10.1136/bmjopen-2018-028631 31175199 PMC6589009

[21] Mastenbroek NJJM. The art of staying engaged: The role of personal resources in the mental well-being of young veterinary professionals. J Vet Med Educ. (2017) 44:84–94. 10.3138/jvme.0216-041R1 28206838

[22] Dyrbye LN West CP Satele D Boone S Tan L Sloan J Shanafelt TD. Burnout among us medical students, residents, and early career physicians relative to the general us population. Acad Med. (2014) 89:443–51. 10.1097/ACM.0000000000000134 24448053

[23] Oxford English Dictionary. Oxford Univ Press. Available online at: https://www.oed.com

[24] Bravata DM Watts SA Keefer AL Madhusudhan DK Taylor KT Clark DM. Prevalence, predictors, and treatment of impostor syndrome: a systematic review. J Gen Intern Med. (2020) 35:1252–75. 10.1007/s11606-019-05364-1 31848865 PMC7174434

[25] Gottlieb M Chung A Battaglioli N Sebok-Syer SS Kalantari A. Impostor syndrome among physicians and physicians in training: a scoping review. Med Educ. (2020) 54:116–24. 10.1111/medu.13956 31692028

[26] Shreffler J Weingartner L Huecker M Shaw MA Ziegler C Simms T. Association between characteristics of impostor phenomenon in medical students and step 1 performance. Teach Learn Med. (2021) 33:36–48. 10.1080/10401334.2020.1784741 32634054

[27] Ladonna KA Ginsburg S Watling C. “Rising to the level of your incompetence”: what physicians' self-assessment of their performance reveals about the imposter syndrome in medicine. Acad Med. (2018) 93:763–8. 10.1097/ACM.0000000000002046 29116983

[28] Perkins DN Salomon G. Transfer of learning. In: Postelthwaite TN Husen T editors. International Encyclopedia of Education. Oxford, UK: Per (1992)

[29] Fraser SW Greenhalgh T. Complexity science: coping with complexity: Educating for capability. Br Med J. (2001) 323:799–803. 10.1136/bmj.323.7316.799 11588088 PMC1121342

[30] ten Cate O Schultz K Frank JR Hennus MP Ross S Schumacher DJ. Questioning medical competence: should the Covid-19 crisis affect the goals of medical education? Med Teach. (2021) 43:817–823. 10.1080/0142159X.2021.1928619 34043931

[31] Ritchhart R Perkins D. Learning to think: the challenges of teaching thinking. In: Holyoak K Morrison R editors. The Cambridge Handbook of Thinking and Reasoning. Cambridge UK: Cambridge University Press (2005). p. 775–802

[32] Bransford JD Brown AL Cocking RR. eds. How People Learn. Washington DC: National Academy Press. (2000).

[33] Vygotsky LS. Mind in Society. The development of higher psychological processes. Cambridge, MA: Harvard University Press. (1978).

[34] ten Cate O Scheele F. Viewpoint: competency-based postgraduate training: can we bridge the gap between theory and clinical practice? Acad Med. (2007) 82:542–547. 10.1097/ACM.0b013e31805559c7 17525536

[35] Busing N Rosenfield J Rungta K Raegele M Warren A Wright B. Smoothing the transition points in canadian medical education. Acad Med. (2018) 93:715–21. 10.1097/ACM.0000000000002072 29166354

[36] Englander R Flynn T Call S Carraccio C Cleary L Fulton TB. Toward defining the foundation of the MD degree: core entrustable professional activities for entering residency. Acad Med. (2016) 91:1352–8. 10.1097/ACM.0000000000001204 27097053

[37] Obeso V Grbic D Emery M Parekh K Phillipi C Swails J. Core entrustable professional activities (EPAs) and the transition from medical school to residency: the postgraduate year one resident perspective. Med Sci Educ. (2021) 31:1813–1822. 10.1007/s40670-021-01370-3 34956699 PMC8651854

[38] ten Cate O. Entrustability of professional activities and competency-based training. Med Educ. (2005) 39:1176–1177. 10.1111/j.1365-2929.2005.02341.x 16313574

[39] Yardley S Westerman M Bartlett M Walton JM Smith J Peile E. The do's, don't and don't knows of supporting transition to more independent practice. Perspect Med Educ. (2018) 7:8–22. 10.1007/s40037-018-0403-3 29383578 PMC5807269

[40] Salzman DH McGaghie WC Caprio TW Hufmeyer KK Issa N Cohen ER. Mastery learning capstone course to teach and assess components of three entrustable professional activities to graduating medical students. Teach Learn Med. (2019) 31:186–94. 10.1080/10401334.2018.1526689 30596271

[41] Wijnen-Meijer M Ten Cate OTJ Van Der Schaaf M Borleffs JCC. Vertical integration in medical school: Effect on the transition to postgraduate training. Med Educ. (2010) 44:272–9. 10.1111/j.1365-2923.2009.03571.x 20444058

[42] Wijnen-Meijer M van den Broek S Koens F ten Cate O. Vertical integration in medical education: the broader perspective. BMC Med Edc. (2020) 509. 10.1186/s12909-020-02433-6 33317495 PMC7737281

[43] Lave J Wenger E. Situated Learning: Legitimate Peripheral Participation. Cambridge,UK: Cambridge University Press. (1991). 10.1017/CBO9780511815355

[44] Dory V Danoff D Plotnick LH Cummings BA Gomez-Garibello C Pal NE. Does educational handover influence subsequent assessment? Acad Med. (2021) 96:118–25. 10.1097/ACM.0000000000003528 32496286

[45] ten Cate O Carraccio C. Envisioning a true continuum of competency-based medical education, training and practice. Acad Med. (2019) 94:1283–1288. 10.1097/ACM.0000000000002687 31460916

[46] Andrews JS Bale JF Soep JB Long M Carraccio C Englander R. Education in Pediatrics Across the Continuum (EPAC): First steps toward realizing the dream of competency-based education. Acad Med. (2018) 93:414–20. 10.1097/ACM.0000000000002020 29023245

[47] Ilgen JS de Bruin ABH Teunissen PW Sherbino J Regehr G. Supported independence: the role of supervision to help trainees manage uncertainty. Acad Med. (2021) 96:S81–6. 10.1097/ACM.0000000000004308 34348381

[48] Turner DA Schwartz A Carraccio C Herman B Weiss P Baffa JM. Continued supervision for the common pediatric subspecialty entrustable professional activities may be needed following fellowship graduation. Acad Med. (2021) 96:S22–8. 10.1097/ACM.0000000000004091 34183598

[49] Schwartz A Borman-Shoap E Carraccio C Herman B Hobday PM Kaul P. Learner levels of supervision across the continuum of pediatrics training. Acad Med. (2021) 96:S42–9. 10.1097/ACM.0000000000004095 34183601

[50] Touchie C Kinnear B Schumacher D Caretta-Weyer H Hamstra SJ Hart D. On the validity of summative entrustment decisions. Med Teach. (2021) 43:780–7. 10.1080/0142159X.2021.1925642 34020576

[51] Smit MP de Hoog M Brackel HJL ten Cate O Gemke RJBJ. A national process to enhance the validity of entrustment decisions for dutch pediatric residents. J Grad Med Educ. (2019) 11:158–64. 10.4300/JGME-D-18-01006 31428274 PMC6697299

[52] ten Cate O Taylor D. The recommended description of an entrustable professional activity: AMEE Guide No. 140. Med Teach. (2021) 43:1106–1114. 10.1080/0142159X.2020.1838465 33167763

[53] Eva KW Regehr G. Self-Assessment in the Health Professions: A Reformulation and Research Agenda. Acad Med. (2005) 80:S46–54. 10.1097/00001888-200510001-00015 16199457

[54] Kruger J Dunning D. Unskilled and unaware of it: How difficulties in recognizing one's own incompetence lead to inflated self-assessments. J Pers Soc Psychol. (1999) 77:1121–34. 10.1037/0022-3514.77.6.1121 10626367

[55] Gignac GE. The association between objective and subjective financial literacy: failure to observe the Dunning-Kruger effect. Pers Individ Dif . (2022) 184: 10.1016/j.paid.2021.111224

[56] Judge TA Bono JE. Relationship of core self-evaluations traits - Self-esteem, generalized self-efficacy, locus of control, and emotional stability - With job satisfaction and job performance: a meta-analysis. J Appl Psychol. (2001) 86:80–92. 10.1037/0021-9010.86.1.80 11302235

[57] Bandura A. Self-efficacy: Toward a unifying theory of behavioral change. Psychol Rev. (1977) 84:191–215. 10.1037/0033-295X.84.2.191 847061

[58] Sawatsky AP O'Brien BC Hafferty FW. Autonomy and developing physicians: reimagining supervision using self-determination theory. Med Educ. (2022) 56:56–63. 10.1111/medu.14580 34091940

